# Optical Dynamic Nuclear Polarization of ^13^C Spins in Diamond at a Low Field with Multi-Tone Microwave Irradiation

**DOI:** 10.3390/molecules27051700

**Published:** 2022-03-04

**Authors:** Vladimir V. Kavtanyuk, Hyun Joon Lee, Sangwon Oh, Keunhong Jeong, Jeong Hyun Shim

**Affiliations:** 1 Quantum Magnetic Imaging Team, Korea Research Institute of Standards and Science, Daejeon 34113, Korea; kavladimir11@gmail.com (V.V.K.); sangwon.oh@kriss.re.kr (S.O.); 2 Radio & Satellite Research Division, Electronics and Telecommunications Research Institute, Daejeon 34129, Korea; hj.lee@etri.re.kr; 3 Korea Military Academy, Seoul 01805, Korea; doas1mind@gmail.com; 4 Department of Medical Physics, University of Science and Technology, Daejeon 34113, Korea

**Keywords:** hyperpolarization, optical dynamic nuclear polarization, nitrogen-vacancy color center, diamond, multi-tone irradiation

## Abstract

Majority of dynamic nuclear polarization (DNP) experiments have been requiring helium cryogenics and strong magnetic fields for a high degree of nuclear polarization. In this work, we instead demonstrate an optical hyperpolarization of naturally abundant ${}^{13}$C nuclei in a diamond crystal at a low magnetic field and the room temperature. It exploits continuous laser irradiation for polarizing electronic spins of nitrogen vacancy centers and microwave irradiation for transferring the electronic polarization to ${}^{13}$C nuclear spins. We have studied the dependence of ${}^{13}$C polarization on laser and microwave powers. For the first time, a triplet structure corresponding to the ${}^{14}$N hyperfine splitting has been observed in the ${}^{13}$C polarization spectrum. By simultaneously exciting three microwave frequencies at the peaks of the triplet, we have achieved ${}^{13}$C bulk polarization of 0.113 %, leading to an enhancement of 90,000 over the thermal polarization at 17.6 mT. We believe that the multi-tone irradiation can be extended to further enhance the ${}^{13}$C polarization at a low magnetic field.

## 1. Introduction

**Figure 1:**
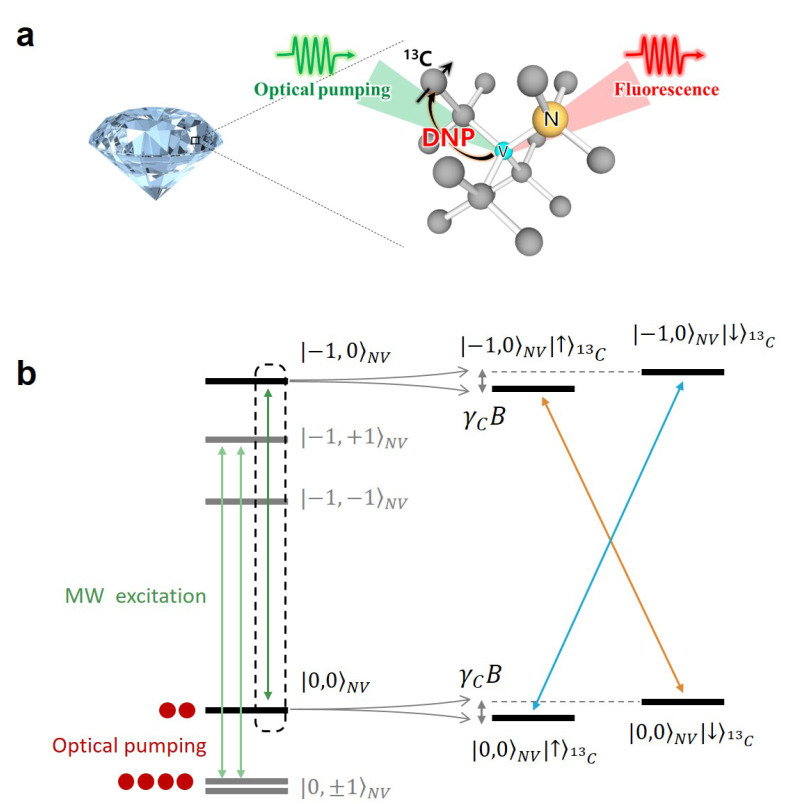
(**a**) The schematic illustration of optical DNP process in diamond. The optically induced polarization in a NV spin transfers to bulk ${}^{13}$C nuclear spins. (**b**) The part of energy level structure associated with NV, ${}^{14}$N and distant ${}^{13}$C spins is given. At a low field, ${}^{13}$C nuclear Zeeman energy $\gamma_{c}B$ is weaker than the ${}^{14}$N hyperfine splitting (2.16 MHz) of NV centers. The arrows indicate possible transitions induced by MW excitation used for the optical DNP.

Dynamic nuclear polarization (DNP) is a technological breakthrough, which can significantly boost the signal-to-noise ratio in nuclear magnetic resonance (NMR) and magnetic resonance imaging (MRI) [1,2,3,4,5,6,7]. Enhanced signal enables extracting substantial amount of information at molecular resolution for a wide range of chemical, biological and physical processes [8,9,10,11,12,13,14,15,16]. Nevertheless, the high cost of conventional DNP instrumentation, based on helium cryogenics and strong magnetic fields, encourages the development of novel DNP techniques. Recently, optical dynamic nuclear polarization has been demonstrated, in which electronic polarization of negatively charged nitrogen-vacancy (NV) centers is transferred to bulk ${}^{13}$C nuclear spins in diamonds (Figure 1a) [17,18,19,20,21,22,23,24,25]. In addition to continuous and simultaneous irradiations of microwave and pump laser, a variety of techniques have been introduced, which include pulsed irradiation of microwave (MW) fields satisfying Hartman–Hahn condition [26], the cross polarization at the level anti-crossing in the ground state [17] and the excited state [18]. For nanodiamonds having significant line broadening due to their random orientation, frequency-swept microwave irradiation [21,22,23,24] has been applied. Hyperpolarization of ${}^{13}$C nuclear spins in diamond may open up new applications in quantum metrology, e.g., a high-field magnetometer [27,28] and a solid-state nuclear spin gyroscope [29]. Moreover, hyperpolarized nanodiamonds have a potential application in molecule-targeted in vivo imaging with the advantage of long spin life times [30,31,32,33,34].

NV-based optical DNP can be performed without a cryogenic apparatus and in the presence of only tens of millitesla. Although the instrumentation may contain elevated magnetic fields, they are essential for the readout of induced nuclear polarizations. The magnetic field in the range of tens of millitesla is easily accessible, but not favorable for an efficient hyperpolarization. Both weak thermal nuclear polarization and short T${}_{1}$ relaxation time [35] are disadvantageous. In addition, the positive and negative nuclear polarization spectra, being a part of solid-state DNP spectrum, are located in close proximity, separated by the order of nuclear resonance frequency [36]. Thus, the overlap of positive and negative nuclear polarizations often occurs and leads to a moderate net polarization. Nevertheless, the nuclear polarization spectrum, which reflects the weak ${}^{13}$C Zeeman splitting, has not been observed in optical DNP studies of diamonds. If ${}^{13}$C resonance frequency is less than ${}^{14}$N hyperfine splitting (2.16 MHz) of NV electronic spin (Figure 1b), the nuclear polarization spectrum would be duplicated three times at intervals of 2.16 MHz. Such triplet structure has not been reported either.

In the present study, we conduct NV-based optical DNP of bulk ${}^{13}$C nuclear spins in diamond at a low field and the room temperature. This hyperpolarization method requires continuous laser and MW irradiations with optimized powers. The applied field for optical DNP is 17.6 mT, which corresponds to ${}^{13}$C resonance frequency of approximately 0.2 MHz. After the hyperpolarization process, a diamond can be shuttled to a center of 6 T superconducting magnet for ${}^{13}$C polarization readout by NMR spectrometer. In the ${}^{13}$C nuclear polarization spectrum, we clearly observe the triplet structure revealing the ${}^{14}$N hyperfine splitting. As anticipated, nitrogen nuclear spin does not participate in the polarization transfer process except for determining the resonance frequency of NV spins through the hyperfine interaction. Such independence can be utilized. Inspired by frequency comb [22], we apply triple microwave frequencies simultaneously and obtain a ${}^{13}$C nuclear polarization of 0.113%, which is about 1.7 times as high as that with single MW frequency excitation.

## 2. Experimental Methods

HPHT-grown diamond crystal with natural abundance of ${}^{13}$C nuclear spins is used in all measurements presented in this paper. NV centers, with a concentration of 1.25 ppm, are created in the diamond via electron irradiation and thermal annealing process.

We have developed a system which includes a low-field region (17.6 mT) for hyperpolarizing ${}^{13}$C in diamonds by DNP, a high-field region (6 T) for all ${}^{13}$C NMR readouts, and a rapid shuttling device for moving the diamond from one region to another in 2 s. From ${}^{13}$C NMR signals at 6 T, enhancement factors and absolute polarization levels can be estimated. ${}^{13}$C NMR acquisition and the timing controls are conducted with a commercial NMR console. (More detailed explanation in the Appendix C).

## 3. Experimental Results

### 3.1. ${}^{13}$C Polarization Spectrum

**Figure 2:**
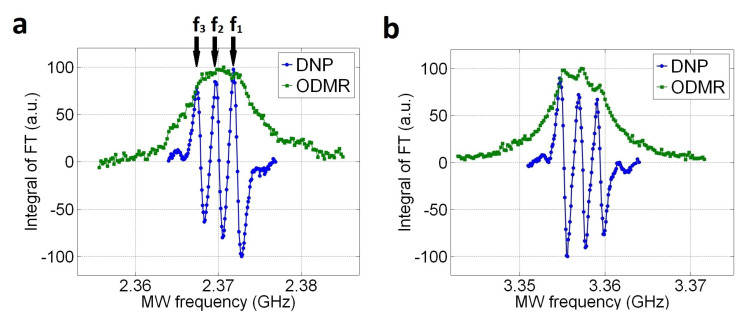
Normalized values of integrated Fourier Transform (FT) signals (blue line) and ODMR signal (green line) are plotted as a function of the MW frequency at 17.6 mT for the transitions of m${}_{s}$ = 0 → m${}_{s}$ = −1 (**a**) and m${}_{s}$ = 0 → m${}_{s}$ = +1 (**b**). Each data point for each MW frequency is measured with the same experimental conditions.

The investigation of the ${}^{13}$C polarization spectrum reveals us the triple structure (Figure 2). Varying the frequency of MW irradiation for optical DNP allows us to record the ${}^{13}$C NMR signal as represented by the blue lines. The green lines illustrate optically-detected magnetic resonance (ODMR) lines of NV center, including the transitions of m${}_{s}$ = 0 → m${}_{s}$ = −1 (Figure 2a) and m${}_{s}$ = 0 → m${}_{s}$ = +1 (Figure 2b). All these measurements are performed through NV centers of 〈111〉 orientation, which is parallel with the direction of magnetic field. Each data point is averaged by 10 measurements. After each NMR scan, a series of 90 pulses are applied to deplete residual ${}^{13}$C polarization ensuring zero polarization for a next measurement. The triplet structures are clearly observed for both NV spin transitions. The ${}^{14}$N (I = 1) nuclear spin of the NV center splits the NV spin levels each into three hyperfine sublevels with an energy splitting of 2.16 MHz, and this explains the triplet shown in Figure 2. Although ODMR does not exhibit the ${}^{14}$N hyperfine splitting, it is clearly visible in the ${}^{13}$C polarization spectra. Notably, the signs of the ${}^{13}$C polarization are identical for both NV spin transitions.

The results in Figure 2 are obtained at a magnetic field of 17.6 mT with a diamond containing natural abundance of ${}^{13}$C nuclear spins. A similar measurement was performed in Ref. [19], where a magnetic field of 18 mT was applied. However, the ${}^{13}$C polarization spectrum showed no triplet structure. Since a diamond of 10% enriched ${}^{13}$C was used in Ref. [19], strong dipolar couplings between NV and proximate ${}^{13}$C spins may dominate over the ${}^{14}$N hyperfine interaction. In Ref. [25], a higher magnetic field of 473 mT was applied, corresponding to a ${}^{13}$C resonance frequency of 5 MHz. In this case, the overlap of three spectra with a separation of 2.16 MHz could wipe the triplet structure, resulting in a single curve.

### 3.2. Optimal Microwave Power and Laser Power

**Figure 3:**
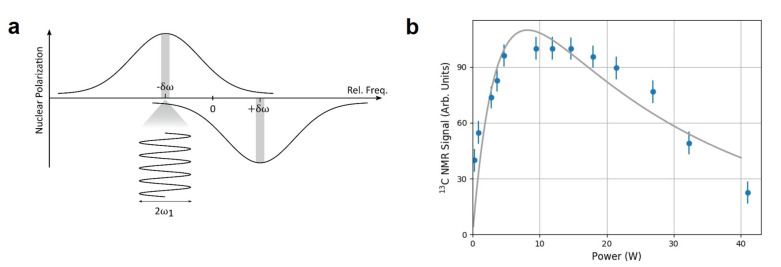
(**a**) The MW excitation, which is on-resonant with the positive polarization, can induce the negative (off-resonant) polarization as well. (**b**) The (normalized) ${}^{13}$C polarization obtained through optical DNP is measured as a function of MW power.

An optimization of the MW power is important for improving ${}^{13}$C polarization. Particularly at a low magnetic field, a strong MW excitation may not be a beneficial. As the separation between positive and negative peaks in the nuclear polarization spectrum is proportional to the nuclear Zeeman splitting [36], the strong MW that is on-resonant with one polarization can also induce the transition of the opposite polarization, which is off-resonant as illustrated in Figure 3a. Then, the opposite polarization will be imposed and eventually lead to a lower net nuclear polarization. Figure 3b shows the ${}^{13}$C nuclear polarization as a function of MW power. The polarization rises initially but decays afterwards as the MW power increases. From the curve in Figure 3, we can determine the optimal MW power, which is near 10 W. The solid line is the fitted curve with two exponents for a guide to eyes. A similar result was reported in Ref. [19], where it was explained by a transition from selective regime to Λ regime when MW becomes stronger.

**Figure 4:**
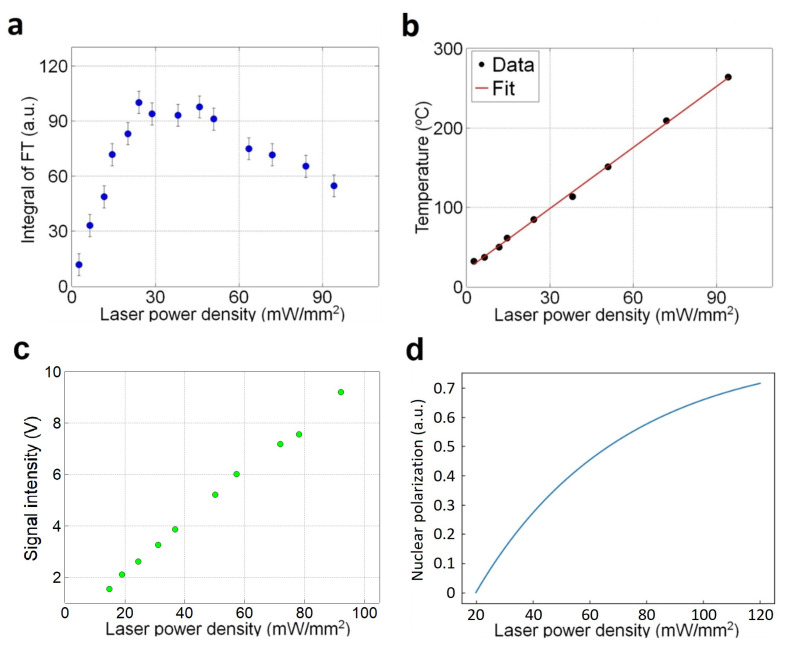
(**a**) The (normalized) ${}^{13}$C polarization obtained through optical DNP is measured as a function of laser power density. (**b**) The variation of diamond’s temperature with increasing the laser power density. (**c**) Fluorescence intensity of NV centers in diamond increases linearly with the laser power density. (**d**) The curve of the ${}^{13}$C polarization expected by using the results in Figure 4b,c.

An optimization of the laser power is also important for improving ${}^{13}$C polarization. Figure 4a shows that the maximum ${}^{13}$C polarization signal is obtained at a laser power density of 30 mW/mm${}^{2}$. After the maximum, the polarization gradually decreases as the laser power increases. Initially, we speculated that the ${}^{13}$C polarization decrease is due to the rise of diamond’s temperature. The temperature of the diamond crystal can be measured from the position of zero-field splitting. In the ODMR spectrum, the peak positions of the two transitions, m${}_{s}$ = 0 → m${}_{s}$ = −1 and m${}_{s}$ = 0 → m${}_{s}$ = +1, can be obtained. Then, their mean leads to the temperature with an aid of the conversion factor, $\frac{dD}{dT}≅-74$ kHz/K [37]. As shown in Figure 4b, the temperature rise is linearly proportional to the laser power density. At the optimal near 30 mW/mm${}^{2}$, the diamond’s temperature is raised above 100 ${}^{\circ }$C. Because the thermal ${}^{13}$C polarization is inversely proportional to temperature, one may attribute the decrease in Figure 4a to the laser-induced heating shown in Figure 4b.

However, that the laser-induced heating alone is an insufficient to explain the ${}^{13}$C polarization decrease. It’s because the NV electronic spin polarization becomes higher with increasing the laser power (or power density). Figure 4c shows the fluorescence intensity from NV centers in the diamond. It increases proportionally to the laser power density. In contrast to Ref. [38], the reduction of the fluorescence when laser power is over a certain level is not observed. This indicates the laser-induced non-radiative process in optical cycle [38] is not activated. Thus, the average polarization of NV centers in the diamond increases linearly within the range of the laser power we apply. The enhancement factor should be proportional to the average NV spin polarization ($P_{\mathrm{NV}}$). ${}^{13}$C polarization ($P_{\mathrm{Hyper}}$) obtained by the optical DNP, then, is determined by the NV spin polarization ($P_{\mathrm{NV}}$) times the ${}^{13}$C thermal polarization ($P_{\mathrm{Thermal}}$) as $P_{\mathrm{Hyper}}\propto P_{\mathrm{NV}}\cdot P_{\mathrm{Thermal}}$. Given a pump laser density $\sigma_{\mathrm{Laser}}$, $P_{\mathrm{NV}}$ and $P_{\mathrm{Thermal}}$ can be expressed as follows

(1) $$\begin{array}{ccc} & & P_{\mathrm{NV}}=\beta \sigma_{\mathrm{Laser}},\\ & & P_{\mathrm{Thermal}}=\frac{c}{300+\alpha \sigma_{\mathrm{Laser}}}, \end{array}$$

in which α and β are the slopes of the curves in Figure 4b,c, respectively. (*c* is a constant from Curie’s law of nuclear para-magnetism.) The resulting curve of $P_{\mathrm{Hyper}}$ is shown in Figure 4d, which is certainly inconsistent with the result in Figure 4a. This discrepancy may indicate the existence of an additional ${}^{13}$C depolarization process in the diamond. Possibly, this depolarization process is thermally activated by the laser-induced heating. And we have measured the nuclear spin-lattice relaxation rate has almost no influence on the decrease of ${}^{13}$C polarization (More details in the Supplementary file).

### 3.3. ${}^{13}$C Hyperpolarization by Multi-Tone MW Frequencies

Since multiple peaks are observed in the ${}^{13}$C polarization spectrum (Figure 2), using several MW sources, we can further enhance the ${}^{13}$C polarization by simultaneous MW irradiations of the frequencies corresponding to those peaks. To estimate the ${}^{13}$C polarization obtained through the optical DNP, we compare the ${}^{13}$C NMR signal with that of thermal polarization at 6 T (300 K). A measurement of ${}^{13}$C thermal polarization, for the diamond, requires at least 4 days, for a better signal-to-noise ratio (SNR), with 400 averages and 20 min interval between each scan. ${}^{13}$C hyperpolarization, with triple MW irradiation, is performed by DNP through −1 state of NV${}^{-}$ in Figure 2a. The diamond is exposed to the pump laser beam with a power density of 25 mW/mm${}^{2}$ and the MW field with a power of 11 W, for 120 s.

**Figure 5:**
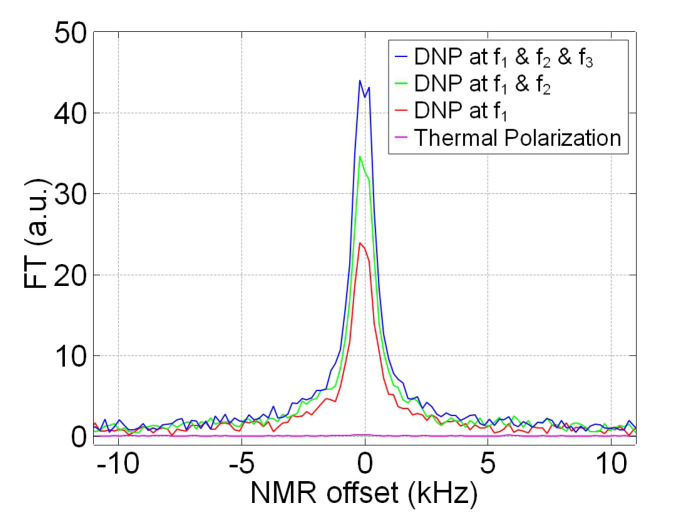
Comparison between thermally polarized ${}^{13}$C at 6 T and hyperpolarized ${}^{13}$C nuclei obtained by the optical DNP at 17.6 mT. All the measurements are performed on the same diamond at room temperature. The pink line represents FT signal obtained from thermally polarized ${}^{13}$C nuclei with 400 averages. The red, green and blue lines represent the FT signals obtained with MW excitations at single ($f_{1}$), double (f${}_{1}$, f${}_{2}$) and triple (f${}_{1}$, f${}_{2}$, f${}_{3}$) frequencies, respectively. The spectral positions of f${}_{1}$, f${}_{2}$ and f${}_{3}$ are indicated in Figure 2a. The ${}^{13}$C polarization increases from 0.068%, through 0.094%, to 0.113%, with the number of MW excitation.

Figure 5 shows results of the multiple MW irradiation. The number of MW frequencies are increased from one (f${}_{1}$) to three (f${}_{1}$, f${}_{2}$, f${}_{3}$). The three MW frequencies are indicated by the arrows in Figure 2a. The ${}^{13}$C polarization increases with the number of MW frequency, from 0.068 % with f${}_{1}$ only to 0.113 % with all three frequencies. However, the enhancement ratio of 1.7 is less than the ideal value of about 2.5 predicted by the summed intensities in the Figure 2a. This may be due to the effect of off-resonant excitation illustrated in Figure 3a. The polarization of 0.113 % corresponds to the enhancement of 90,000 over the in situ thermal polarization at 17.6 mT and the diamond’s temperature, as the calculation shown in the Appendix B.

## 4. Conclusions

We have demonstrated the optical DNP results obtained with a diamond crystal of natural abundance of ${}^{13}$C nuclei at room temperature and a low magnetic field of 17.6 mT. A triplet structure in ${}^{13}$C nuclear polarization spectrum has been observed, in which the splitting of 2.16 MHz attributes to ${}^{14}$N hyperfine interaction in NV centers. We have reached 0.113% of bulk ${}^{13}$C nuclear spin polarization. The optimal values of laser power density and MW power have been characterized. In addition, multi-tone MW excitation has been adopted. Simultaneous irradiation of three MW frequencies, matched to the peak frequencies in the ${}^{13}$C nuclear spectrum, results in an improvement of 1.7 times. The number of microwave frequencies in the multi-tone irradiation scheme could be extended to further enhance the ${}^{13}$C polarization at a low magnetic field. 

## Figures and Tables

**Figure A1:**
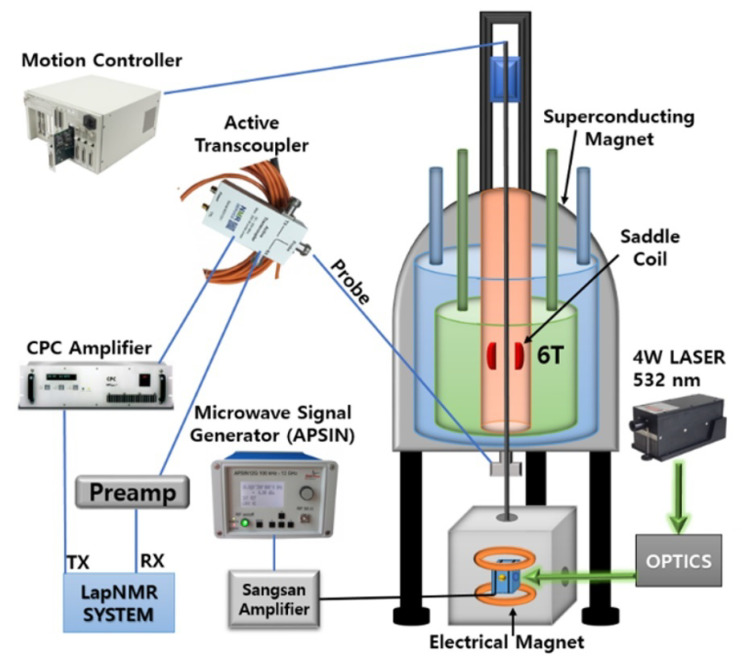
Full experimental setup.

**Figure A2:**
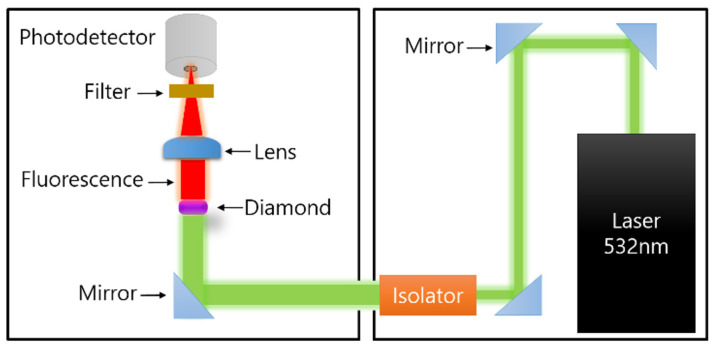
Schematic of the laser optical setup.

## Data Availability

Not applicable.

## Supplementary Materials

The following supporting information are available online.

## Sample Availability

Samples of the compounds are not available from the authors.

## Appendix A. Diamond Sample

All the experimental results are measured from one industrial diamond (ElementSix) having a weight of 42 mg. It is synthesized by the high-pressure high-temperature method, which is, then, electron irradiated at 1 MeV and annealed at about 800 ${}^{\circ }$C. The diamond contains about 50 ppm of P${}_{1}$ centers, 1.25 ppm of NV centers, and the natural abundance of ${}^{13}$C. The spin concentrations in the diamond are estimated by performing electron spin resonance (ESR). The peak intensities in ESR spectra are compared to that from a diamond crystal having a known P${}_{1}$ concentration. The diamond is 〈111〉 surface-oriented.

## Appendix B. Estimation of ^13^C Nuclear Polarization and Enhancement Factor

The hyperpolarization of ${}^{13}$C in the diamond is conducted at $B_{\mathrm{EM}}$ = 17.6 mT. The thermal polarization of the same diamond is obtained at $B_{SM}$ = 6 T. The thermal polarization (*P*${}_{\mathrm{Thermal}}$) is used for estimating the ${}^{13}$C polarization (*P*${}_{\mathrm{Hyper}}$):

(A1) $$P_{\mathrm{Hyper}}=\frac{S_{\mathrm{Hyper}}}{S_{\mathrm{Thermal}}}*P_{\mathrm{Thermal}},$$

(A2) $$P_{Thermal}=\frac{\hbar \gamma B_{0}}{2kT_{R}},$$

where $S_{\mathrm{Hyper}}$ is the integral of FT signal from a hyperpolarized ${}^{13}$C nuclei in the diamond. $S_{\mathrm{Thermal}}$ is the integral of FT signal measured from thermally polarized ${}^{13}$C nuclei in the diamond. $T_{R}$ is the room temperature.

The enhancement factor (ε) is about 90,000 over in situ thermal equilibrium polarization and can be estimated as follows:

(A3) $$\varepsilon =\frac{S_{\mathrm{Hyper}}}{S_{\mathrm{Thermal}}}*\frac{B_{\mathrm{SM}}}{B_{\mathrm{EM}}}*\frac{T_{L}}{T_{R}},$$

where $T_{L}$ is diamond’s temperature under laser irradiation during DNP process (Figure 4b).

## Appendix C. Experimental Setup

Our experimental setup is a sophisticated system capable of hyperpolarizing ${}^{13}$C in diamonds using DNP method and reading out polarizations by NMR method. This section thoroughly describes the experimental setup (Figure A1) and a purpose of each hardware.

### Appendix C.1. Superconducting Magnet and NMR Hardware

The magnetic field of a superconducting magnet (OXFORD) is used for all NMR readouts. It has a diameter of 70 cm and a height of 97 cm. The magnet produces a homogeneous magnetic field of about 6 T in its central region. There is a cylindrical cavity with a diameter of about 5.2 cm in the center of magnet through its entire height. A handmade saddle coil is installed inside the cavity in homogeneous region for detecting polarization signals from samples. The coil is connected to a NMR probe (Figure A1), which is LC circuit made of parallel and serial connected variable capacitors. By manipulating the capacitors, we can match the impedance and set a required resonance frequency for the saddle coil (∼64.237 MHz for ${}^{13}$C). The probe is subsequently connected to an active-transcoupler and to a commercial spectrometer LapNMR from Tecmag. The purpose of transcoupler is to manage switching between transmitted signals from the spectrometer and received NMR signals from the saddle coil. The spectrometer is controlled by TNMR software, which allows us to set all required parameters for generating proper RF signals and acquire NMR signals. Transmitted RF signals, from LapNMR, are increased by CPC 9T100M power amplifier. NMR signals, captured by the saddle coil, are increased by two +35 dB amplifiers from the NMR service.

### Appendix C.2. MW Hardware

One of the aspects in successful hyperpolarization of ${}^{13}$C in a diamond is the application of MW field with enough power. In our experiments, MW signals are produced by APSIN12G and amplified by Sungsan TS2458BBP0 with a maximum power output of 50 W. A handmade Helmholtz coil, with a diameter of 13 mm, is used to generate MW fields (Figure A1). A circulator (PE83CR1004) with a 50 W termination is used to protect the amplifier from reflected MW power. The MW coil is installed inside of an electrical Helmholtz magnet (Figure A1), which is powered by a high precision DC power supply (KEITHLEY 2280S-32-6). The electrical magnet is installed right below the superconducting magnet and used for optical DNP. It provides a decent homogeneity and an easy access to variable magnetic fields.

### Appendix C.3. Optical Configuration

Our laser produces vertically polarized 532 nm green light with a maximum power output of 4 W and a beam diameter of 2 mm. The delivery of the laser light to diamonds is performed by two separated optical setups (Figure A2). The left setup locates inside a shielding box, where we perform optical DNP on the diamonds. The box shields ambient magnetic fields. The right setup locates outside of the shielding box and includes three mirrors, an isolator and the laser itself.

The isolator is used to protect from reflected lights and to increase the beam size to a diameter of 5 mm matching the diamond’s size. The long pass filter is used to exclude lights except the red light emitting by the diamond. The lens, with focal length of 25.4 mm, guides the emitted fluorescence, from diamonds, to a photodetector with a diameter of about 2 mm.

## Appendix D. Experimental Procedure

Before each DNP experiment, ODMR measurement is performed at a specific magnetic field. The diamond is irradiated with a wide range of MW sweep (up to 1.5 GHz) and with 532 nm laser light. An emitted red light from a diamond is then captured by a photo-detector. A data from the detector can be seen on a computer using a Labview program, where we can define exact MW frequencies corresponding to the states and estimate a magnetic field.

All DNP experiments, in this work, are performed at the magnetic field of about 17.6 mT, aligned with the one of four NV center’s orientations in diamond crystals. ${}^{13}$C nuclear spins, in the diamond, are hyperpolarized for over 100 s with continuous MW and laser (532 nm) irradiations. After ${}^{13}$C nuclei are hyperpolarized, the diamond is shuttled 80 cm within 2 s, by a high-precision motion controller Newport XPS-RL, into the superconducting magnet, where the polarization is detected by the saddle coil. Then, the ${}^{13}$C NMR signal at the resonance frequency of 64.237 MHz is transmitted to LapNMR console. Single NMR acquisition takes 5 ms and then the diamond is sent back to the low-field region within 2 s for repetitions. For a better SNR, the hyperpolarized ${}^{13}$C NMR signal is typically averaged 10 times. For multi-tone MW excitation, three MW frequencies are synthesized by three separate sources and then combined into the MW coil.
