# Supplementary material for: Optical Dynamic Nuclear Polarization of 13C Spins in Diamond at a Low Field with Multi-Tone Microwave Irradiation
Source: Molecules. 2022 Mar 4;27(5):1700. doi: 10.3390/molecules27051700 (PMC8911784; doi:10.3390/molecules27051700)
Supplement: Supplementary file 1 [file molecules-27-01700-s001.zip › molecules-1553220-supplementary.pdf]

Article

Optical dynamic nuclear polarization of ^{13}C spins in diamond at a low field with multi-tone microwave irradiation - Supplementary Material

Vladimir V. Kavtanyuk [1], Hyun Joon Lee[2], Sangwon Oh [1], Keunhong Jeong [3] and Jeong Hyun Shim [1,4,*]

1 Quantum Magnetic Imaging Team, Korea Research Institute of Standards and Science, Daejeon 34113, Republic of Korea
2 Radio & Satellite Research Division, Electronics and Telecommunications Research Institute, Daejeon 34129, Republic of Korea
3 Korea Military Academy, Seoul 01805, Republic of Korea
4 Department of Medical Physics, University of Science and Technology, Daejeon 34113, Republic of Korea
* Correspondence: jhshim@kriss.re.kr

1. T_1 comparison at different laser power

Spin-lattice relaxation time (T_1) may significantly change as the diamond temperature increases by the laser-induced heating. But, it is hard to access directly to the temperature dependence of T_1. Instead, one can expect that the polarization time should reflect the spin-lattice relaxation time. Thus, we have measured polarization build-up curves at different laser power densities used for the optical DNP (Fig. 1). As shown in Fig. 4 (b) in the main text, the diamond temperature increases linearly with the laser power density. We have found that the polarization times are nearly identical up to the power density of 54 mW/mm^2. This result indicates that the nuclear spin-lattice relaxation rate has almost no influence on the decrease of ^{13}C polarization shown in Fig. 4 (a) in the main text, and it supports our claim of the existence of additional depolarization process that is thermally activated by the laser-induced heating.

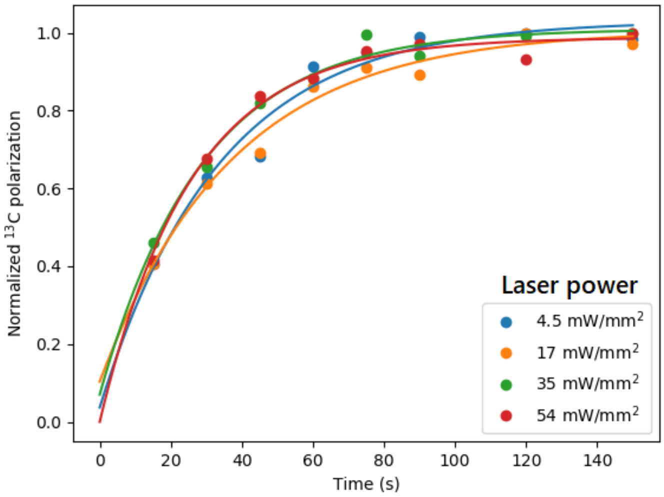

Figure S1. Normalized ^{13}C polarization vs build-up time at different laser power density.
